# Tumor Mutation Burden, Immune Cell Infiltration, and Construction of Immune-Related Genes Prognostic Model in Head and Neck Cancer

**DOI:** 10.7150/ijms.51064

**Published:** 2021-01-01

**Authors:** Ai-Min Jiang, Meng-Di Ren, Na Liu, Huan Gao, Jing-Jing Wang, Xiao-Qiang Zheng, Xiao Fu, Xuan Liang, Zhi-Ping Ruan, Tao Tian, Yu Yao

**Affiliations:** Department of Medical Oncology, The First Affiliated Hospital of Xi'an Jiaotong University, Xi'an, Shaanxi, People's Republic of China.

**Keywords:** Head and neck squamous cell carcinoma (HNSCC), Tumor mutation burden (TMB), Immune cell infiltration, Immune-related genes (IRGs), TCGA

## Abstract

**Background:** Head and neck squamous cell carcinoma (HNSCC) is the sixth most common malignancy worldwide, and the prognosis of HNSCC remains bleak. Numerous studies revealed that the tumor mutation burden (TMB) could predict the survival outcomes of a variety of tumors.

**Objectives:** This study aimed to investigate the TMB and immune cell infiltration in these patients and construct an immune-related genes (IRGs) prognostic model.

**Methods:** The expression data of 546 HNSCC patients were obtained from The Cancer Genome Atlas (TCGA) database. All patients were divided into high- and low- TMB groups, and the relationship between TMB and clinical relevance was further analyzed. The differentially expressed genes (DEGs) were identified using the R software package, limma. Functional enrichment analyses were conducted to identify the significantly enriched pathways between two groups. CIBERSORT algorithm was adopted to calculate the abundance of 22 leukocyte subtypes. The IRGs prognostic model was constructed via the multivariate Cox regression analysis.

**Results:** Missense mutation and single nucleotide variants (SNV) were the most predominant mutation types in HNSCC. *TP53*, *TTN*, and *FAT1* were the most frequently mutated genes. Patients with high TMB were observed with worse survival outcomes. The functional analysis of TMB associated DEGs showed that the identified DEGs mainly involved in spliceosome, RNA degradation, proteasome, and RNA polymerase pathways. We observed that macrophages, T cells CD8, and T cells CD4 memory were the most commonly infiltrated subtypes of immune cells in HNSCC. Finally, an IRGs prognostic model was constructed, and the AUC of the ROC curve was 0.635.

**Conclusions:** Our results suggest that high TMB is associated with poor prognosis in HNSCC patients. The constructed model has potential prognostic value for the prognosis of these individuals, and it needs to be further validated in large-scale and prospective studies.

## Introduction

Head and neck squamous cell carcinoma (HNSCC) mainly including nasopharyngeal carcinoma, oropharyngeal carcinoma, hypopharyngeal carcinoma, and larynx carcinoma, is the sixth most common malignancy worldwide 1. More than 600,000 new cases of HNSCC are diagnosed each year, and the mortality of HNSCC is staggering at 40%, accounting for 3.6% of cancer-related deaths 1, 2. Although significant improvements have been made in screening, diagnosis, and precise management in recent years, the prognosis of HNSCC remains bleak, with an approximately 5-year survival rate of 60% for patients with locoregionally advanced disease 3, 4.

In recent years, the clinical application of immune checkpoint inhibitors (ICIs) has demonstrated promising response rates in various malignant tumors, approving that it could play an antitumor role by reversing immunodeficiency and activating the immune cells 5-10. Although numerous studies have shown that recurrent and metastatic HNSCC patients could benefit from immunotherapy 11-15, most of them are resistant to ICIs, with a 13-18% overall response rate 16. As recent studies suggested, limited immune cell infiltration in the tumor microenvironment (TME), reduced tumor immunogenicity, and co-expression of inhibitory immune-related genes were involved in the resistance phenomenon of immunotherapy in various malignant tumors, including HNSCC 4, 16. Furthermore, accumulating evidence indicates that the emerging biomarkers such as tumor mutation burden (TMB), neoantigens, and microsatellite instability (MSI) are associated with immunotherapy response 17, 18.

With the rapid development of transcriptome-sequencing analysis and the extensive application of bioinformatic datasets, a growing number of studies paid close attention to the prognostic role of TMB, immune cell infiltration, and immune-related genes across different types of malignancy. However, only limited data are available in HNSCC. Therefore, we conducted the present study to comprehensively explore the prognostic value of TMB and the association with immune cell infiltration in HNSCC and to construct immune-related genes prognostic model using The Cancer Genome Atlas (TCGA) dataset.

## Materials and methods

### Raw data download and analysis

The gene expression profile, clinical profile, and somatic mutation data of 546 HNSCC patients (tumor samples, 502 cases; normal samples, 44 cases) were obtained from the TCGA database (https://tcgadata.nci.nih.gov/tcga/). The R software package, maftools, was used to summarize and visualize the Masked Somatic Mutation data 19.

### Calculation of TMB value

TMB was defined as the total number of somatic gene coding errors, base substitutions, insertions, or deletions detected per megabyte bases of tumor tissue 20. According to the previous study, the estimated TMB value for each sample was defined as the total mutation frequency/the length of the human exon (38 Mb) 21. Besides, all synonymous mutations were excluded for calculation of TMB.

### Relationship between TMB value and prognosis and clinical features

The HNSCC samples were divided into low- and high-TMB groups by the median value (2.079) of TMB. We further merged the TMB value with their corresponding survival variables by matching id number of each patient, and the Kaplan-Meier analysis in R package was adopted to evaluate the relationship between TMB and prognosis in HNSCC. Subsequently, the R package was used to assess the relationship of TMB values with clinical characteristics.

### Differentially expressed genes and functional pathways analysis

The differentially expressed genes (DEGs) were identified using the R software package, limma. |log2 FC| > 1.0, and false discovery rate (FDR) < 0.05 were used as a filter. Besides, the R software package, pheatmap was adopted to generate heatmap plot to visualize the difference. Then, the R software packages, clusterProfiler, org.Hs.eg.db, enrichplot, and ggplot2 were applied to conduct the Gene Ontology (GO) and Kyoto Encyclopedia of Genes and Genomes (KEGG) pathway enrichment analyses 22. The significantly enriched pathways were considered with both *p*- and *q*-value of < 0.05. Gene set enrichment analysis (GSEA) was conducted using TMB level as the phenotype label. We chose c2.cp.kegg.v6.2.symbols.gmt gene sets as reference gene sets and GSEA 4.0 software was applied to data analysis. NOM *p* < 0.05 and FDR *q* < 0.25 were used as the criteria of significant enrichment pathways.

### Immune cell infiltration

The R software package, CIBERSORT, was adopted to calculate the abundance of 22 leukocyte subtypes in 502 tumor samples, and a threshold of *P*-value <0.05 was considered as cut-off criteria 23. Furthermore, Wilcoxon rank-sum test was performed to analyze the difference of immune cells infiltration in low- and high- TMB groups, with the R software package, vioplot was used to visualize the difference.

### Construction of immune-related genes prognostic model

A total of 2,498 immune-related genes (IRGs) were downloaded from the immunology database and analysis portal (Immport) (https://www.immport.org/home), and the differentially expressed immune-related genes (DEIRGs) were identified using the R software package, VennDiagram. We then merged the identified DEIRGs with their corresponding survival information by matching id number. The R software package, survival, was applied to perform univariate and multivariate Cox regression analyses. The hazard ratios (HRs) and 95% confidence intervals (95%CIs) of IRGs in the prognostic model were calculated. Besides, the survival difference between the low- and high- expression groups of IRGs was compared via Kaplan-Meier analysis. A *P*-value < 0.05 was considered with a statistical difference. We then conducted a multivariate Cox regression analysis to obtain the risk score of each patient and the coefficients of identified hub IRGs. The risk score was calculated based on the following formula 24:


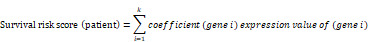


In the formula, '*k*' represents the total number of the immune genes in the prognostic model. '*Gene i*' represents the *i^th^* selected immune gene, and '*coefficient (gene i)*' represents the coefficient of the immune gene in multivariate Cox analysis. Furthermore, according to the median value of risk score, the HNSCC patients were classified into low- and high- risk groups, and the Kaplan-Meier analysis was performed to compare the survival differences between the two groups. Finally, the Receiver Operating Characteristic (ROC) curve was performed to evaluate the accuracy of the constructed IRGs prognostic model.

### Relationship between copy number variation of the IRGs and immune cell infiltration

Tumor IMmune Estimation Resource (TIMER) web server is a comprehensive resource for systematically analyzing immune infiltrates in various malignancies. The abundances of six immune cells infiltration (B cells, CD4+ T cells, CD8+ T cells, Neutrophils, Macrophages, and Dendritic cells) were estimated by the TIMER algorithm 25. We further investigated the relationship between copy number variation (CNV) of the IRGs in the prognostic model and immune cell infiltration using the TIMER 2.0 web server (https://cistrome.shinyapps.io/timer/). Wilcoxon rank-sum tests were used to compare the differences of immune cell subsets in each mutation status and normal infiltration level. Furthermore, box plots were adopted to show the distribution of immune cell subsets among each mutation groups in HNSCC patients. A *P*-value < 0.05 was considered with statistic significant.

### Statistical analysis

All statistical analysis was performed using R software (version 3.6.1) and Bioconductor (https://www.bioconductor.org/). The R software package, limma, was used to differential analysis. The 'survival' package was adopted to the Cox regression model construction. The survival difference was evaluated and visualized using Kaplan‐Meier survival curves, and the association was tested via log-rank tests. One-way ANOVA, unpaired two-tailed t-test, and Tukey's multiple-comparison post-hoc test were utilized to evaluate the relationship of TMB levels and clinical characteristics of HNSCC as appropriate. Wilcoxon rank-sum test and Kruskal-Wallis test were used to non-parametric statistical tests as appropriate. The ROC curve was adopted to assess the predictive ability of the prognostic model, with an AUC value > 0.60 was considered as acceptable for predictions, and an AUC > 0.75 was regarded as has the excellent predictive ability 26, 27.

## Results

### Mutations in HNSCC patients

**Figure 1:**
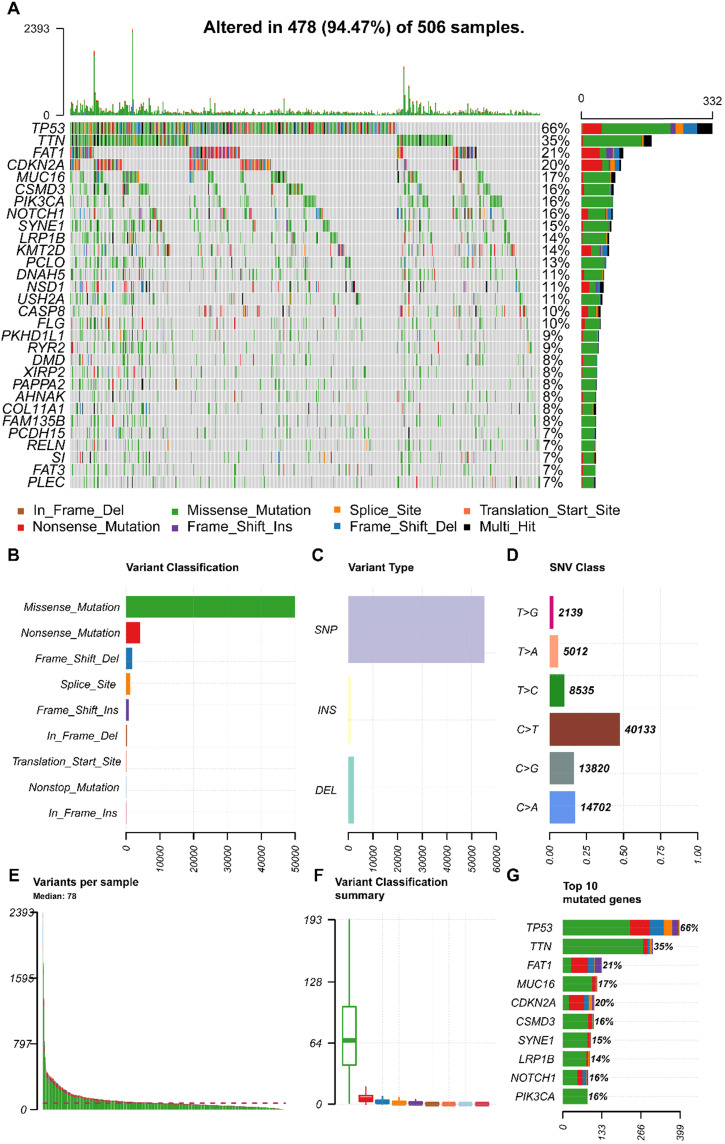
** The landscape of mutation profiles in HNSCC patients. (A)** Waterfall plot of the top 30 mutated genes in the TCGA HNSCC Cohort; **(B, C, D)** Classification of mutation types according to different categories; **(E, F)** TMB in specific samples; **(G)** the top 10 mutated genes in HNSCC. HNSCC, head and neck squamous cell carcinoma; TCGA, The Cancer Genome Atlas; TMB, tumor mutation burden.

**Figure 2:**
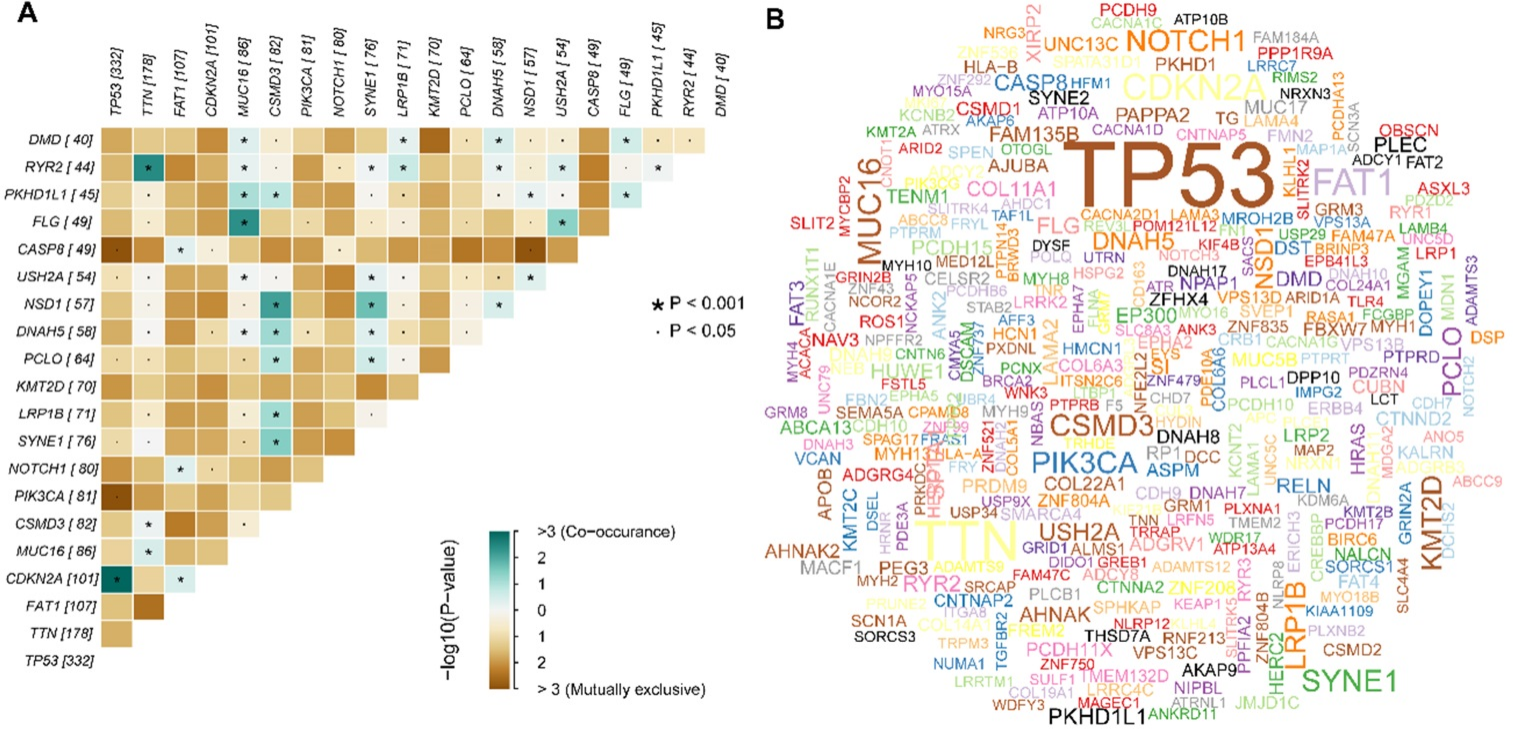
** Summary of the mutation information with statistical calculations. (A)** The coincident and exclusive associations across mutated genes; **(B)** Genecloud plot of the mutated frequencies.

We obtained the mutation profile of HNSCC patients from the TCGA database, and the R software package, maftools, was applied to present the results. It showed that 94.47% (478) of patients occurred somatic mutation, with missense mutation and single nucleotide variants (SNV) being the most predominant mutation types (**Figure 1A, B, C**). Besides, C > T transversion was the most primary type of single nucleotide variants (SNV) in HNSCC (**Figure 1D**). Furthermore, we observed that *TP53* (66%), *TTN* (35%), *FAT1* (21%), *CDKN2A* (20%), *MUC16* (17%), *CSMD3* (16%), *NOTCH1* (16%), *PIK3CA* (16%), *SYNE1* (15%), and *LRP1B* (14%) were the top 10 mutated genes in HNSCC (**Figure 1G**). We also summarized the coincident and exclusive relationships among the mutated genes in **Figure 2A**, with turquoise representing co-occurrence mutation and brown representing mutually exclusive mutation. Besides, we also used the Genecloud plot to show the mutated frequencies of other genes (**Figure 2B**).

### Analysis of TMB and clinical relevance in HNSCC

**Figure 3:**
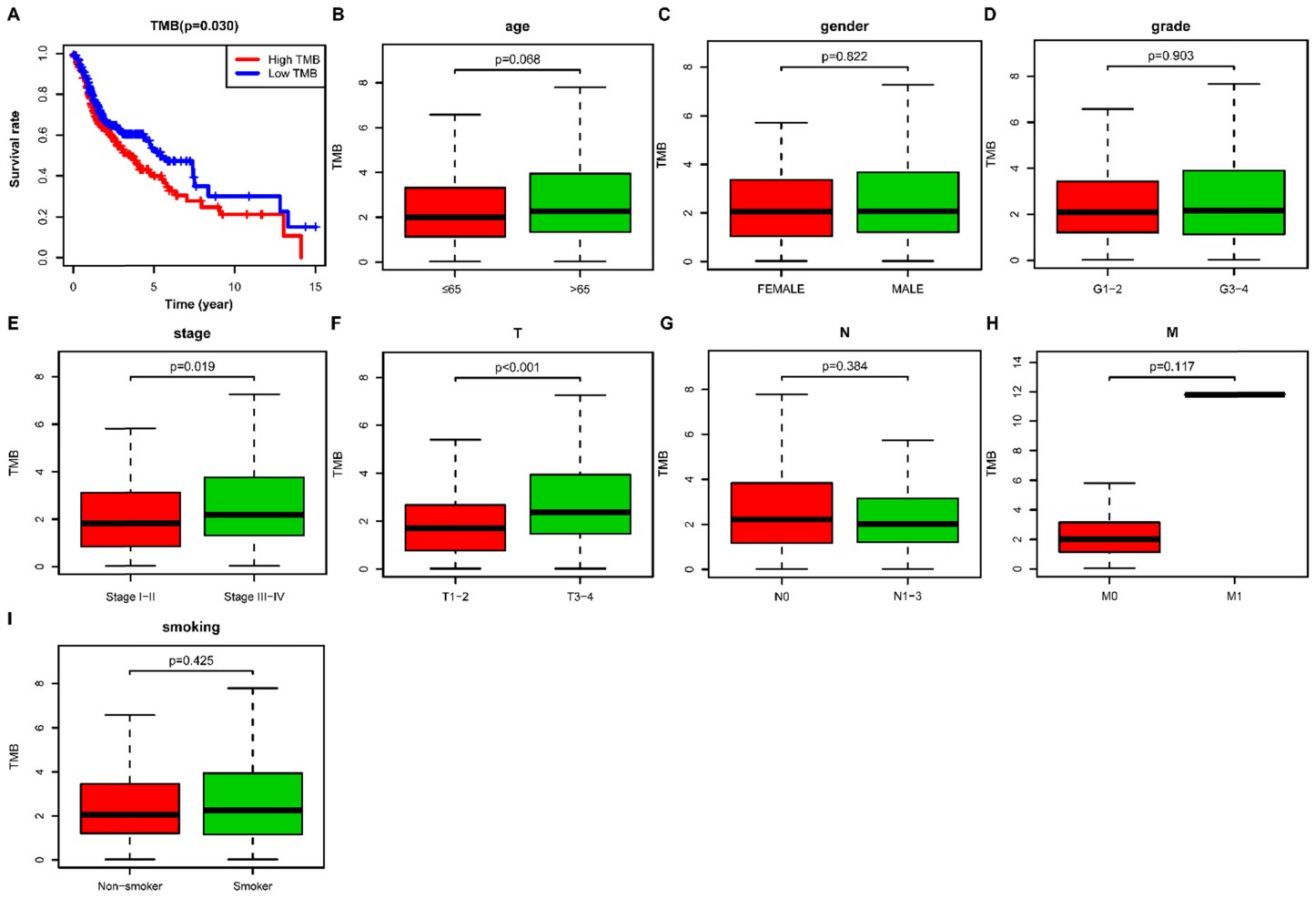
** Correlation of TMB with prognosis and clinicopathological characteristics in HNSCC. (A)** Kaplan-Meier curves of overall survival of the high- and low-TMB groups; **(B)** Wilcox test for HNSCC patients stratified by age;** (C)** Wilcox test for HNSCC patients stratified by gender; **(D)** Wilcox test for HNSCC patients stratified by grade; **(E)** Wilcox test for HNSCC patients stratified by stage; **(F)** Wilcox test for HNSCC patients stratified by AJCC-T stage; **(G)** Wilcox test for HNSCC patients stratified by AJCC-N stage;** (H)** Wilcox test for HNSCC patients stratified by AJCC-M stage; **(I)** Wilcox test for HNSCC patients stratified by smoking history. TMB, tumor mutation burden; HNSCC, head and neck squamous cell carcinoma.

We then calculated the estimated TMB value of each sample and divided them into low- and high- TMB groups according to the median value of TMB. The result of Kaplan-Meier survival analysis indicated that high- TMB status was significantly associated with decreased overall survival (OS) in HNSCC patients (*P*= 0.030) (**Figure 3A**). We further investigated the relationship of TMB value and clinicopathological characteristics in HNSCC, and it revealed that high- TMB levels significantly correlated with the advanced clinical stage (*P* = 0.019) (**Figure 3E**) and higher AJCC-T stage (*P* < 0.001) (**Figure 3F**). However, we did not observe significant association between TMB and age (**Figure 3B**), gender (**Figure 3C**), grade (**Figure 3D**), AJCC-N stage (**Figure 3G**), AJCC-M stage (**Figure 3H**), and smoking history (**Figure 3I**).

### Comparison of gene expression profiles and functional enrichment analysis between low- and high-TMB groups

**Figure 4:**
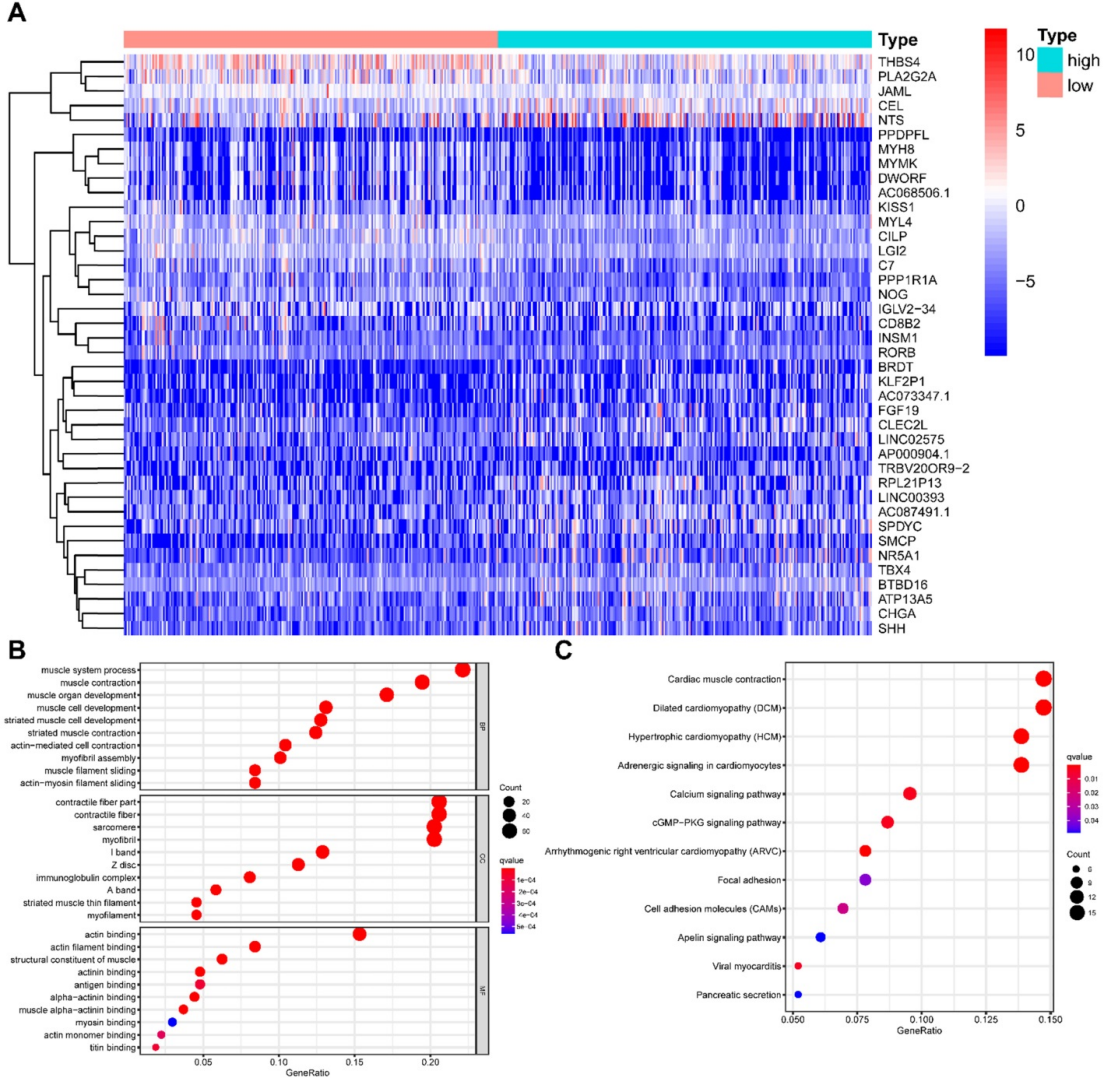
** Comparisons of gene expression profiles in high- and low-TMB samples. (A)** Top 40 DEGs were shown in the heatmap plot;** (B)** Functional analysis of the top 10 enriched biological processes (BPs), cell composition (CC), and molecular function (MF) of GO analysis; **(C)** KEGG enrichment diseases analysis. TMB, tumor mutation burden; DEGs, differentially expressed genes.

**Figure 5:**
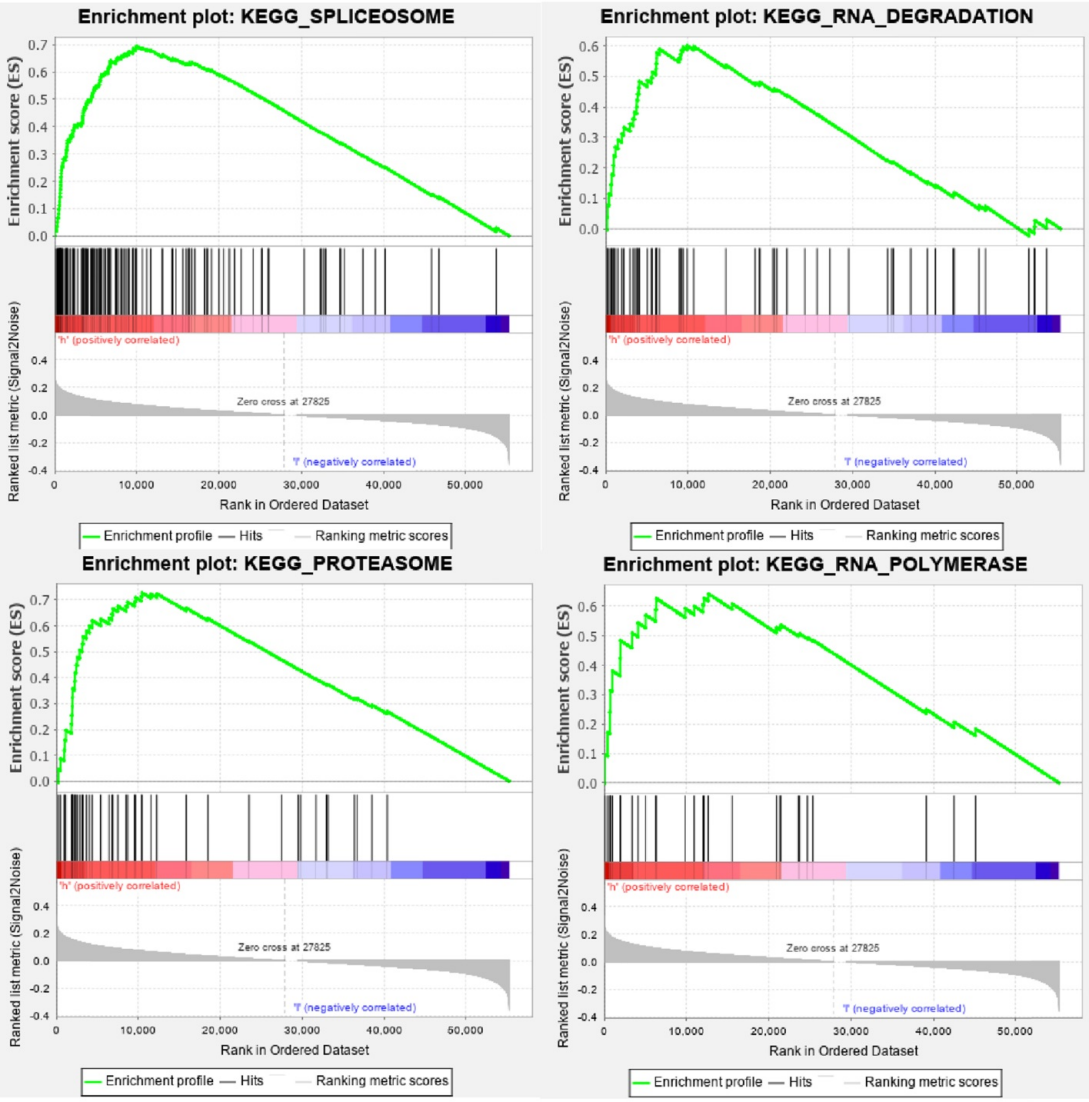
** GSEA enrichment analysis of TMB-related DEGs.** DEGs, differentially expressed genes.

All HNSCC patients were divided into two groups according to the TMB value, and a total of 429 TMB associated DEGs were identified using |log2 FC| > 1.0 and FDR < 0.05 as cut-off criteria. Of these, 136 were up-regulated genes, and 293 were down-regulated genes.** Figure 4A** presented the top 20 DEGs of each group using a hierarchical clustering heatmap. We further performed GO and KEGG enrichment analyses to explore the most common biological processes and pathways involved in these DEGs. The results of GO enrichment analysis showed that these DEGs were primarily enriched in muscle system process, muscle contraction, and muscle organ development **(Figure 4B)**, and the results of KEGG enrichment analysis indicated that these DEGs were primarily enriched in cardiac muscle contraction, dilated cardiomyopathy (DCM), and hypertrophic cardiomyopathy (HCM)** (Figure 4C)**. Furthermore, we performed the GSEA enrichment analysis according to the TMB level, and it showed that high- TMB can activate spliceosome, RNA degradation, proteasome, and RNA polymerase pathways (**Figure 5**).

### Immune cell infiltration in HNSCC

**Figure 6:**
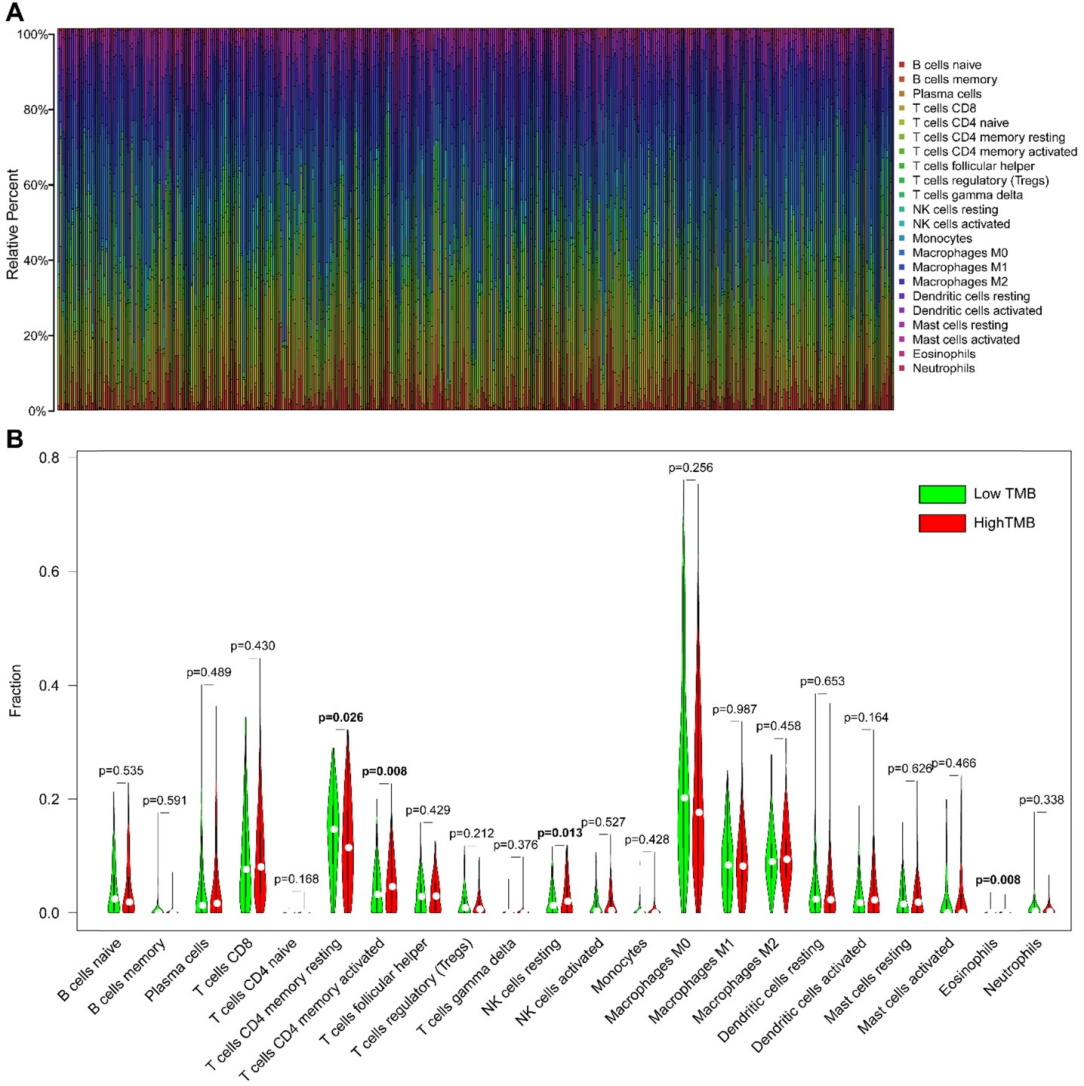
** Comparisons of 22 subsets of immune cells infiltration between low- and high-TMB groups. (A)** Summary of each type of immune cells in HNSCC samples;** (B)** Differential analysis of immune cells infiltration between high- and low-TMB groups. TMB, tumor mutation burden; HNSCC, head and neck squamous cell carcinoma.

The CIBERSORT algorithm was used to estimate the abundance of 22 subtypes of immune cells in 502 HNSCC patients, and a threshold of *P*-value <0.05 was considered as cut-off criteria. There were 421 patients selected to perform the immune cell infiltration analysis, and the relative abundance of 22 immune cells was summarized in **Figure 6A.** We compared the distribution of these immune cells between low- and high- TMB groups in HNSCC. The results showed that T cells CD4 memory activated, NK cells resting, and Eosinophils were significantly infiltrated in the high-TMB group, while T cells CD4 memory resting were significantly infiltrated in low-TMB groups (**Figure 6B).**

### Construction of IRGs prognostic model for HNSCC

**Figure 7:**
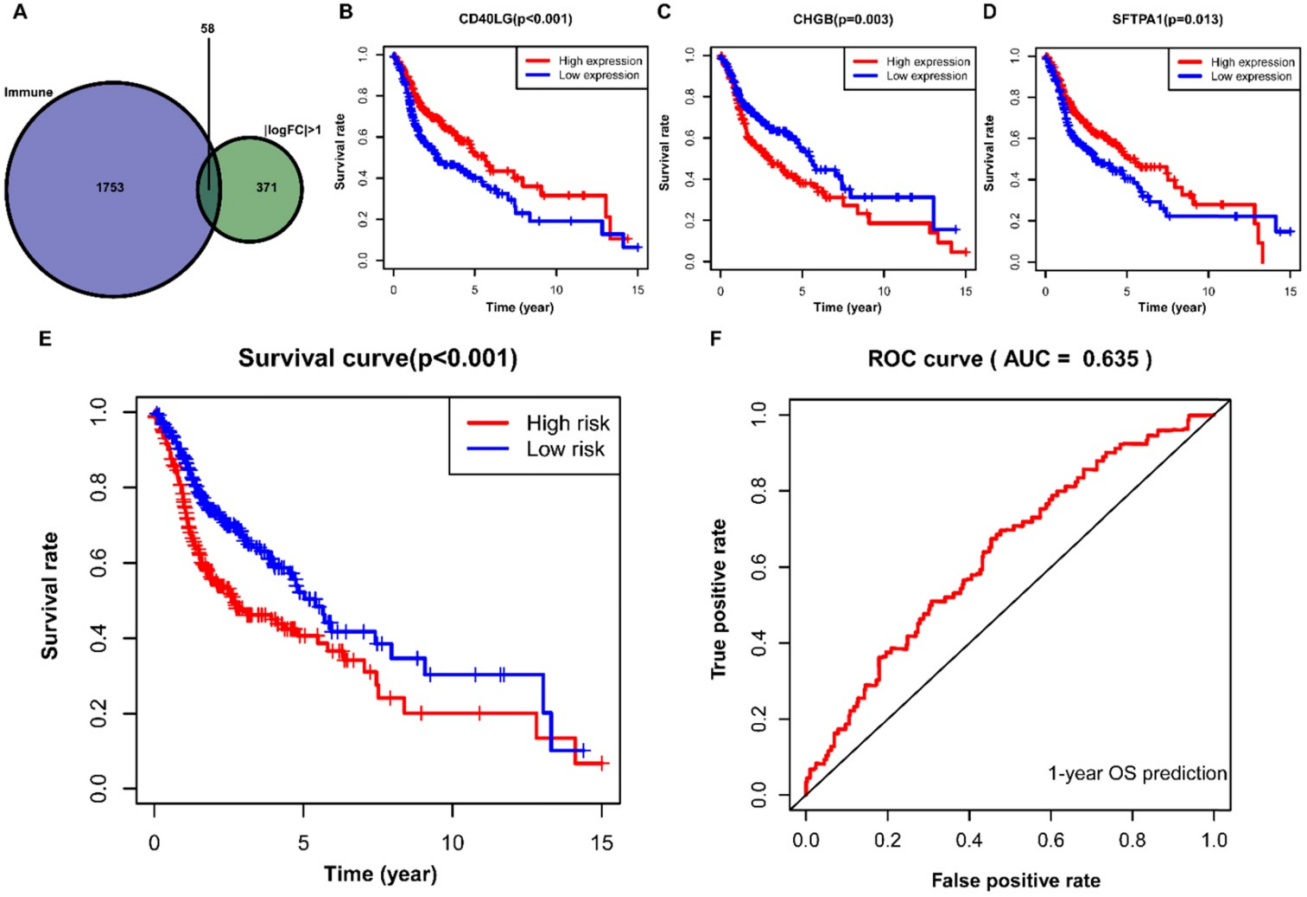
** Construction of IRGs prognostic model for HNSCC. (A)** The identification of IRGs;** (B, C, D)** IRGs identified via multivariate Cox regression analysis;** (E, F)** Construction and assessment of IRGs prognostic model for HNSCC. IRGs, immune-related genes; HNSCC, head and neck squamous cell carcinoma.

A total of 58 differentially expressed IRGs were identified via the R software package, VennDiagram (**Figure 7A**). Besides, we merged these immune-related genes and their corresponding survival information by matching id number. First, we used univariate Cox regression analysis to identify potential targeted IRGs, and four IRGs (*SFTPA1, CD40LG, IGHG2*, and *CHGB*) were identified as candidate genes for prognostic model construction. Ultimately, we obtained three optimal IRGs (*SFTPA1, CD40LG,* and *CHGB*) for inclusion in the prognostic model via multivariate Cox regression analysis. Of these, the high expression of *CD40LG* and *SFTPA1* were significantly correlated with favorable prognosis in HNSCC, while the high expression of *CHGB* was associated with poor prognosis (**Figure 7B, C, D**). We further calculated the risk score of each patient using the estimated coefficient of each IRGs in the prognostic model to evaluate the significance of these IRGs in predicting the prognosis of HNSCC patients. The risk score was calculated based on the following computational formula:

Survival risk score = (0.0751×expression of *SFTPA1*)+(-0.6628×expression of *CD40LG*)+(0.0019×expression of *CHGB*)

Then, we divided the HNSCC patients into low-risk (n=245) and high-risk (n=245) groups according to the median value of the calculated risk score, and we conducted the Kaplan-Meier analysis to compare the survival difference between the two groups. It showed that patients in the high-risk group were associated with decreased OS (*P* < 0.001) (**Figure 7E**). We then performed a ROC curve to evaluate the predictive accuracy of the model for a one-year OS in HNSCC patients, and the AUC value was 0.635 in the prognostic model (**Figure 7F**).

### Association of CNV of the prognostic IRGs and immune cell infiltration

**Figure 8:**
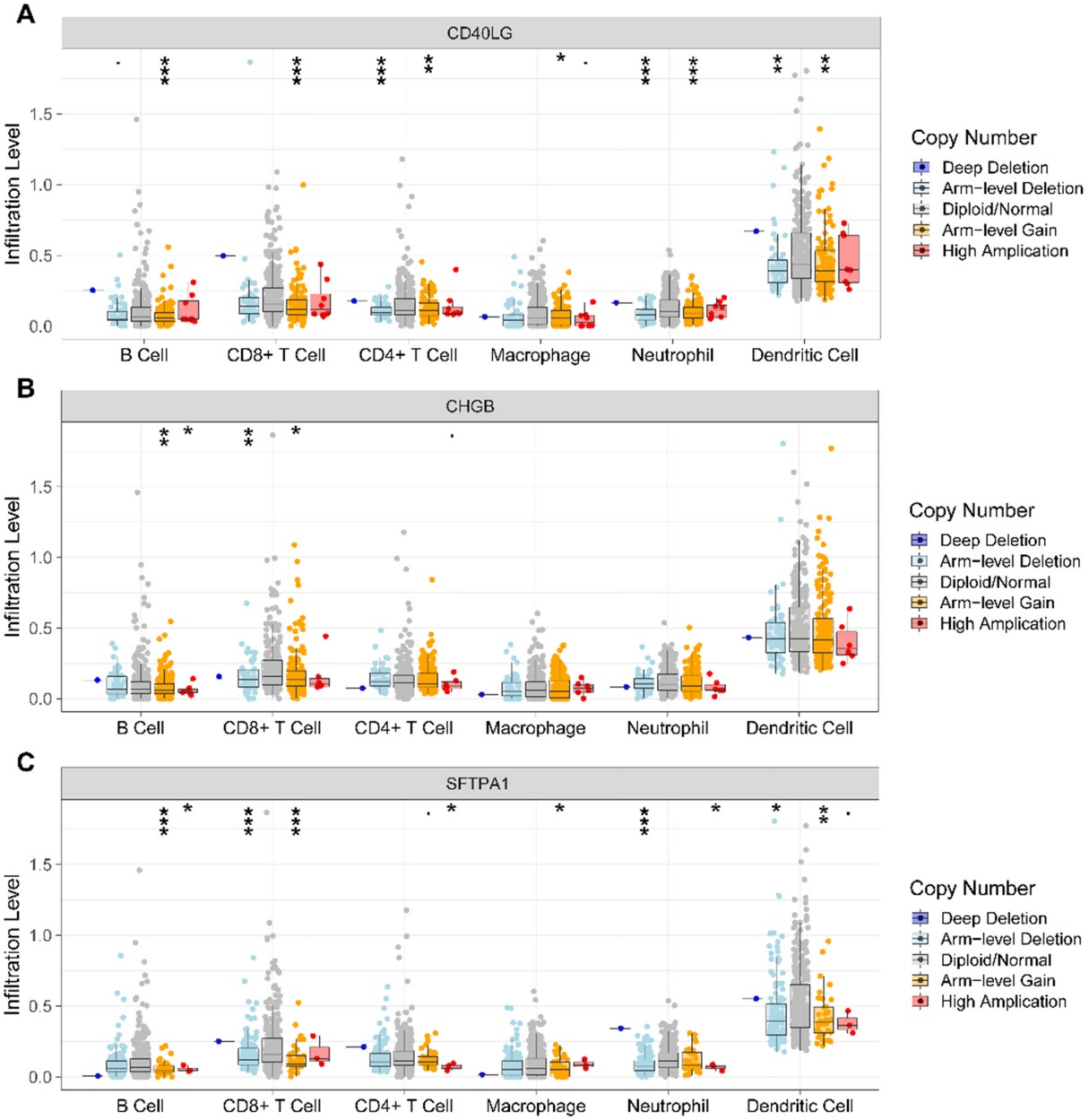
** Analysis of CNV of the IRGs and immune cell infiltration via TIMER. (A)**. *CD40LG*; **(B)** *CHGB*; **(C)** *SFTPA1*. CNV, copy number variation; IRGs, immune-related genes.

We further investigated the association of CNV of the IRGs in the prognostic model and immune cell infiltration using the TIMER web server. The results revealed that compared with normal copy number, other forms of deletion or amplification of copy number may inhibit the infiltration of immune cells to varying degrees (**Figure 8A, B, C**).

## Discussion

HNSCC is a heterogeneous malignancy of the upper aerodigestive tract, and it is also one of the most common causes of cancer-related death worldwide 1, 2. Although significant advances have been made in the screening, diagnosis, and treatment of HNSCC in recent decades, especially the clinical application of immunotherapy, it remains a poor prognosis 3, 4. Numerous studies have highlighted that immune cell infiltration, tumor immunogenicity, and immune-related genes were involved in the resistance phenomenon of immunotherapy in HNSCC 4, 16. Previous studies demonstrated that oncological patients with high TMB are predisposed to have a promising response to immunotherapy 21, 28. However, a recent multicentre retrospective study revealed that high TMB was significantly associated with reduced overall survival (OS) in HNSCC patients treated with definitive chemoradiation 17. Herein, we conducted the current study to explore the prognostic value of TMB and the association with immune cell infiltration in HNSCC and to construct immune-related genes prognostic model using the TCGA dataset.

In the present study, we explored the landscape of TMB in HNSCC patients, showing that *TP53*, *TTN*, and *FAT1* were the most predominant mutated genes. *TP53* is one of the famous tumor suppressors inhibiting tumor occurrence and development by regulating proliferation, apoptosis, angiogenesis, and DNA repair 29. Numerous studies suggest that *TP53* is frequently mutated in various cancers and is correlated with reduced OS 18. Furthermore, it showed that HNSCC patients with *TP53* mutations have bleak prognosis than *TP53*-wildtype HNSCCs 30. P53 protein plays an essential role in tumor suppression and genome stability maintenance, and a recent study revealed that the activation of P53 in TME could enhance antitumor immunity response 31. *FAT1* is a member of the Drosophila fat gene family, and it can inhibit cell proliferation and tumor growth by binding ß-catenin and subsequently decreasing ß-catenin translocation to the nucleus 32. Furthermore, it promotes cell proliferation and migration by interacting with Ena/VAPS and Scribble 33. Therefore, the role of *FAT1* in carcinogenesis is controversial, being reported as both tumor suppressive and oncogenic 33, 34.

We further explored TMB and its clinical relevance in HNSCC. The finding of our study indicated that HNSCC patients with high- TMB status were significantly correlated with the poor OS, which was compatible with the result of a multicentre retrospective study conducted in Germany 17. Zhang et al. also reported that high- TMB was an unfavorable prognostic factor in clear cell renal cell carcinoma 35. However, most previously published studies indicated that high- TMB was associated with favorable survival outcomes in different types of malignancies, including melanoma, non-small cell lung cancer (NSCLC), bladder cancer, and HER2-positive refractory metastatic breast cancer 21, 36, 37. Subsequently, we ferreted out TMB-associated DEGs and investigated their potential biological functions using GO, KEGG, and GSEA enrichment analyses. We observed that high TMB-associated DEGs mainly involved in spliceosome, RNA degradation, proteasome, and RNA polymerase pathways. The spliceosome is a powerful molecular machine consisting of several nuclear protein complexes that cycle on and off of pre-mRNA during intronic splicing 38. Alternative pre-mRNA splicing plays a vital role in establishing and maintaining human cell types by permitting the expression of multiple transcript isoforms from a single gene 39. However, dysregulation of alternative splicing is related to the initiation, progression, and therapeutic response of cancer 39. The proteasome regulates cell cycle, transcription, signaling, trafficking, and protein quality control by degrading most cellular proteins 40. The degradation of the proteasome is pivotal in all cells and organisms, and the misregulation of proteasome function is associated with diverse human diseases, including cancer 40.

The tumor-infiltrating immune cells in TME are relatively associated with carcinogenesis, progression, angiogenesis, and metastasis across different types of cancer. We further investigated the infiltration abundance of 22 subsets of immune cells in HNSCC samples using the CIBERSORT algorithm. In the present study, we observed that macrophages (M0, M1, and M2), T cells CD8, and T cells CD4 memory (resting and activated) were the most commonly infiltrated subtypes of immune cells in HNSCC regardless of TMB status. Similar results were reported in a bioinformatic study conducted by Song et al. 41. Macrophages are essential components of the immune system and present different genotypes and functions in different TME. Tumor-associated macrophages (TAMs) are essential regulators of carcinogenesis, and TAMs frequently exhibit an M2 phenotype that is associated with worse prognosis by promoting angiogenesis and invasion in tumors 42. T cells CD8 plays an antitumoral role under hypoxia conditions by differentiating into lytic effector cells 43. The high abundance of CD8 T cell infiltration is positively associated with favorable survival outcome in HNSCC, and it could predict the future survival rates of patients 4. However, CD4 T cells in TME have different subsets and may have different functions. The results of a previous study revealed that cancer cells induce interleukin-22 (IL-22) production from T cells CD4 memory via interleukin-1 (IL-1) to promote tumor growth 44. To the best of our knowledge, no study investigated the relationship between immune cell infiltration and TMB in HNSCC so far. In our study, we identified that T cells CD4 memory activated, NK cells resting, and Eosinophils were primarily infiltrated in the high-TMB group, while T cells CD4 memory resting were primarily infiltrated in the low-TMB group. It suggests that TMB is closely related to the TME.

In the present study, we further identified 58 differentially expressed IRGs and constructed an IRGs prognostic model via univariate and multivariate Cox regression analyses. In the prognostic model, we identified that the high expression of *CD40LG* and *SFTPA1* were significantly correlated with favorable survival outcome in HNSCC, while the high expression of *CHGB* was associated with poor prognosis. As reported, *CD40LG* is a member of the tumor necrosis factor (TNF) family, mainly expressed on the surface of T cells CD4 activated and providing the necessary signal for immune response 45. It was reported that the overexpression of *CD40LG* on T lymphocytes was observed in human and murine lupus 45. Takezaki et al. reported that *SFTPA1* mainly involved in the development of idiopathic pulmonary fibrosis by promoting necroptosis of alveolar epithelial type II cells via JNK-mediated up-regulation of Ripk3 46. *CHGB* is initially identified in pheochromocytoma, and it encodes a tyrosinesulfated secretory protein (CHGB protein) that is expressed in endocrine cells and neurons. In a recent study, Stenman et al. indicated that the overexpressed *CHGB* was associated with progressive behavior and poor prognosis in pheochromocytomas and abdominal paragangliomas 47. In the prognostic model, we found that patients with high-risk scores had worse survival outcomes. Considering the AUC of this model was only 0.635, we think it has potential prognostic value in HNSCC patients. Furthermore, it needs to be validated in large-scale and prospective studies.

Copy number variation (CNV) is a form of genomic structural variation that can lead to abnormal copy numbers of one or more parts of DNA, including amplification, gain, loss, and deletion. It was reported that CNV plays a vital role in the carcinogenesis of a variety of malignant tumors 48. In our study, we explored the relationship of CNV of IRGs and immune cell infiltration using TIMER. The results showed that compared with normal copy number, other forms of deletion or amplification of copy number may inhibit the infiltration of immune cells in HNSCC, which was compatible with the results of a previous study conducted by Zhang et al. 35. To the best of our knowledge, this is the first study that comprehensively analyzed TMB, immune cell infiltration, and constructed an IRGs prognostic model in HNSCC. However, there are also several inevitable limitations in our study. First, there was no relevant basic experiment to detect the expression of the identified prognostic associated IRGs in cell lines or clinical samples; second, we did not further explore the relationship of immune cell infiltration and the identified IRGs in the model. Therefore, the findings in our study need to be validated in large-scale and prospective studies.

## Conclusions

In summary, our study provides a systematic analysis of TMB and immune cell infiltration in HNSCC patients and constructs an IRGs prognostic model. The results suggest that high TMB is associated with worse prognosis in HNSCC patients. Macrophages, T cells CD8, and T cells CD4 memory are the most commonly infiltrated subtypes of immune cells in HNSCC. Furthermore, we also identified three TMB-related IRGs and constructed a prognostic model. Further studies are warranted to verify the clinical utility of this prognostic model for HNSCC.
